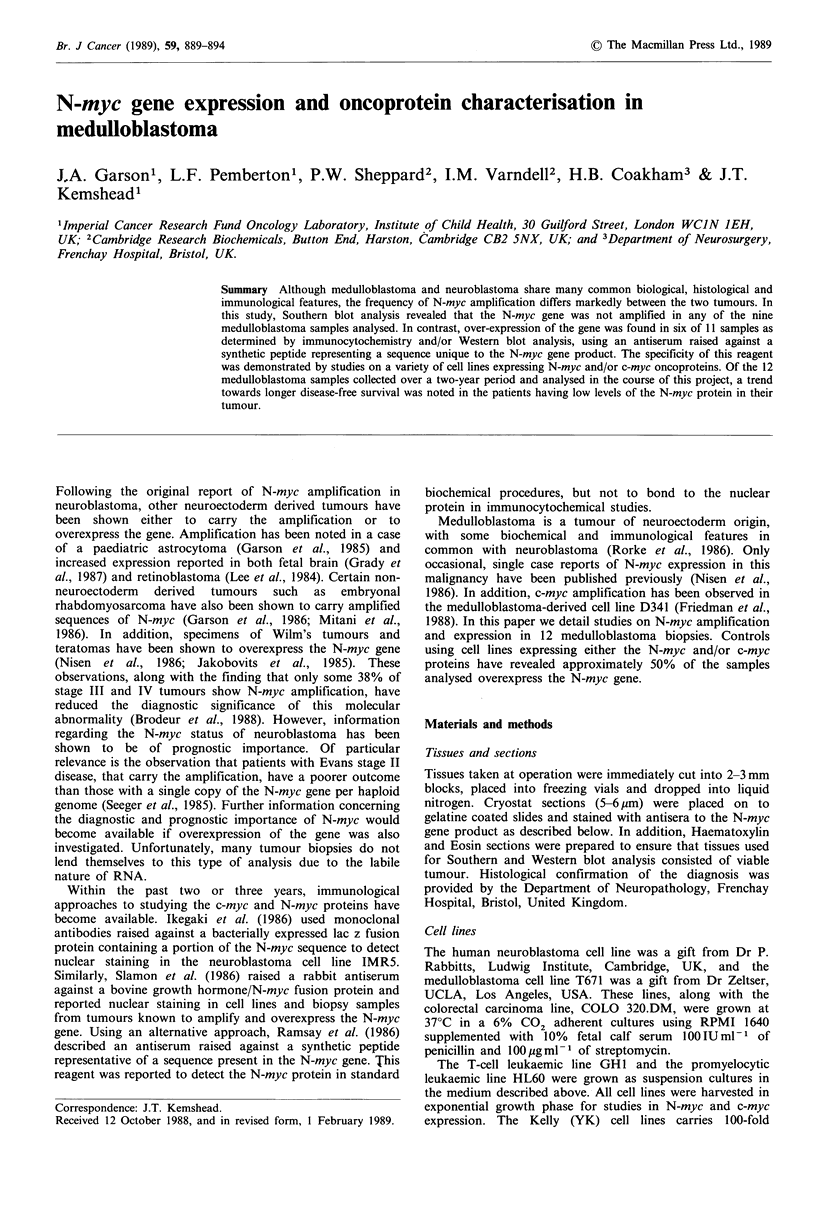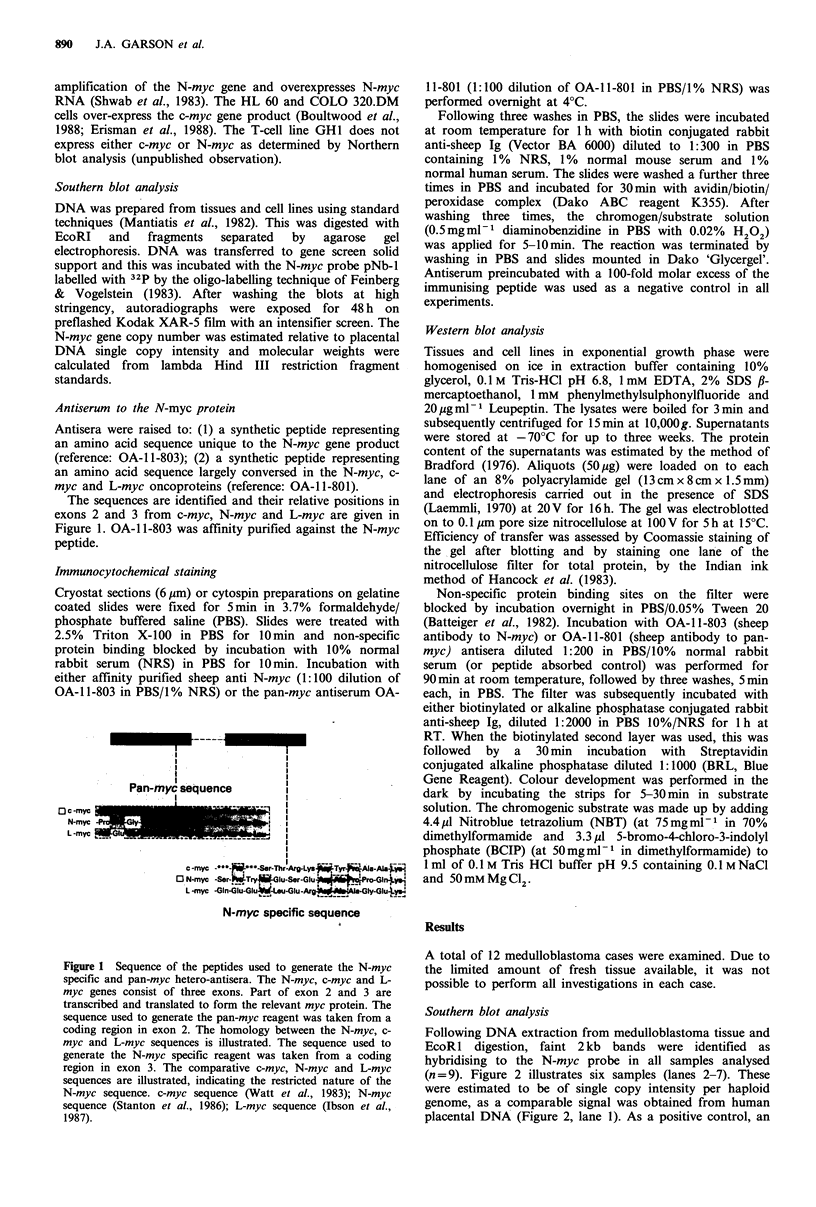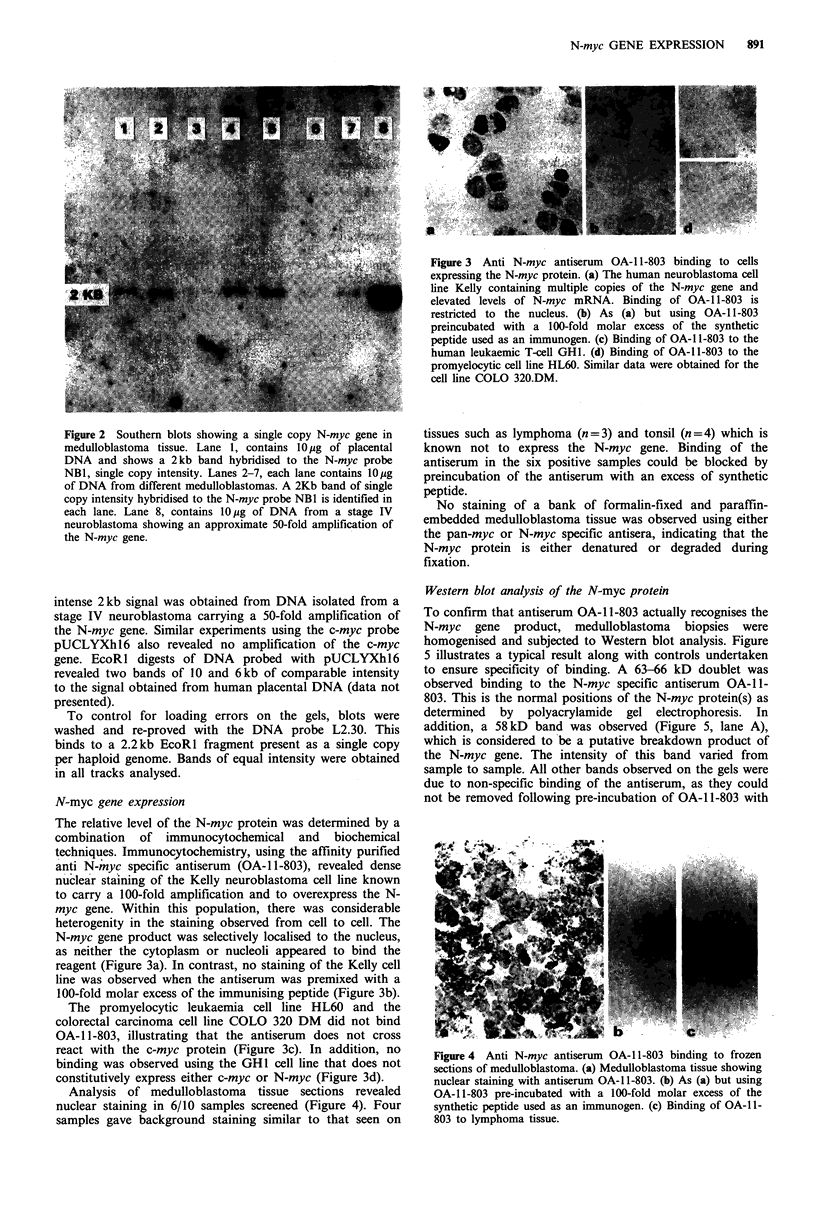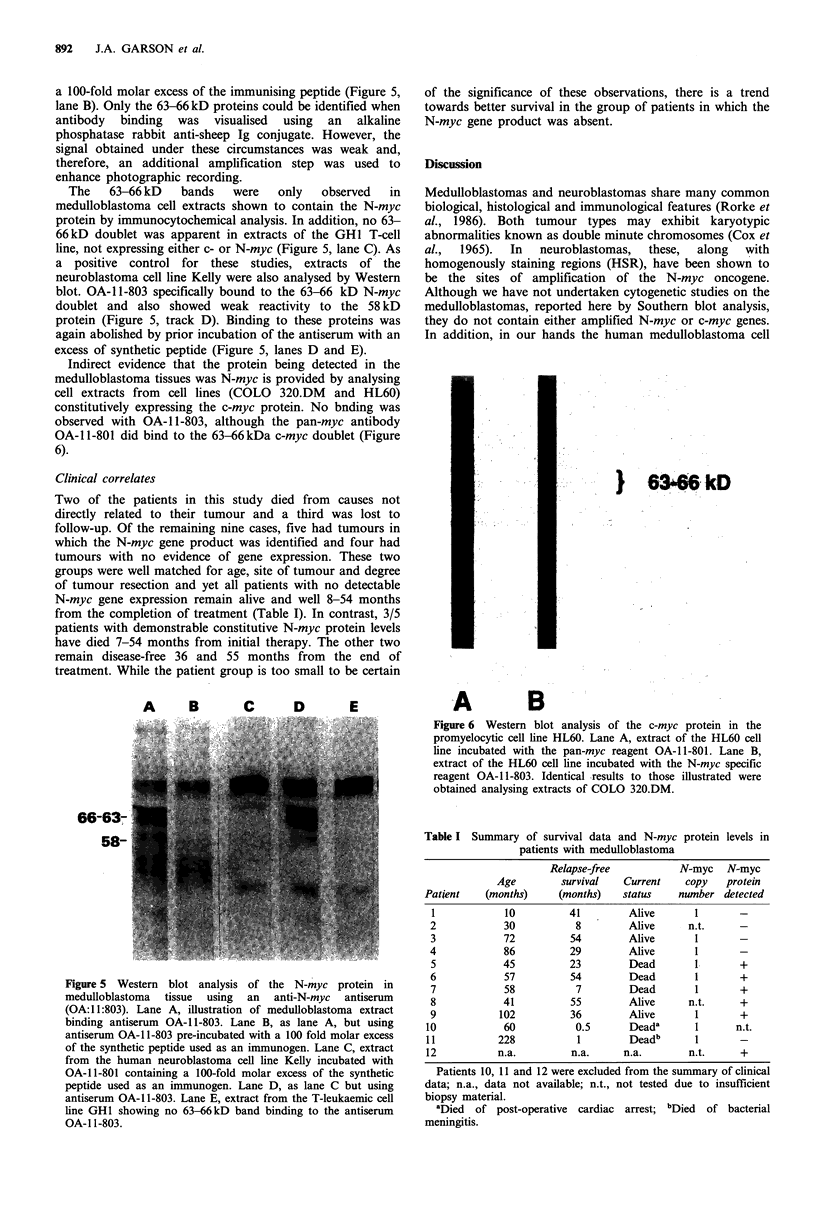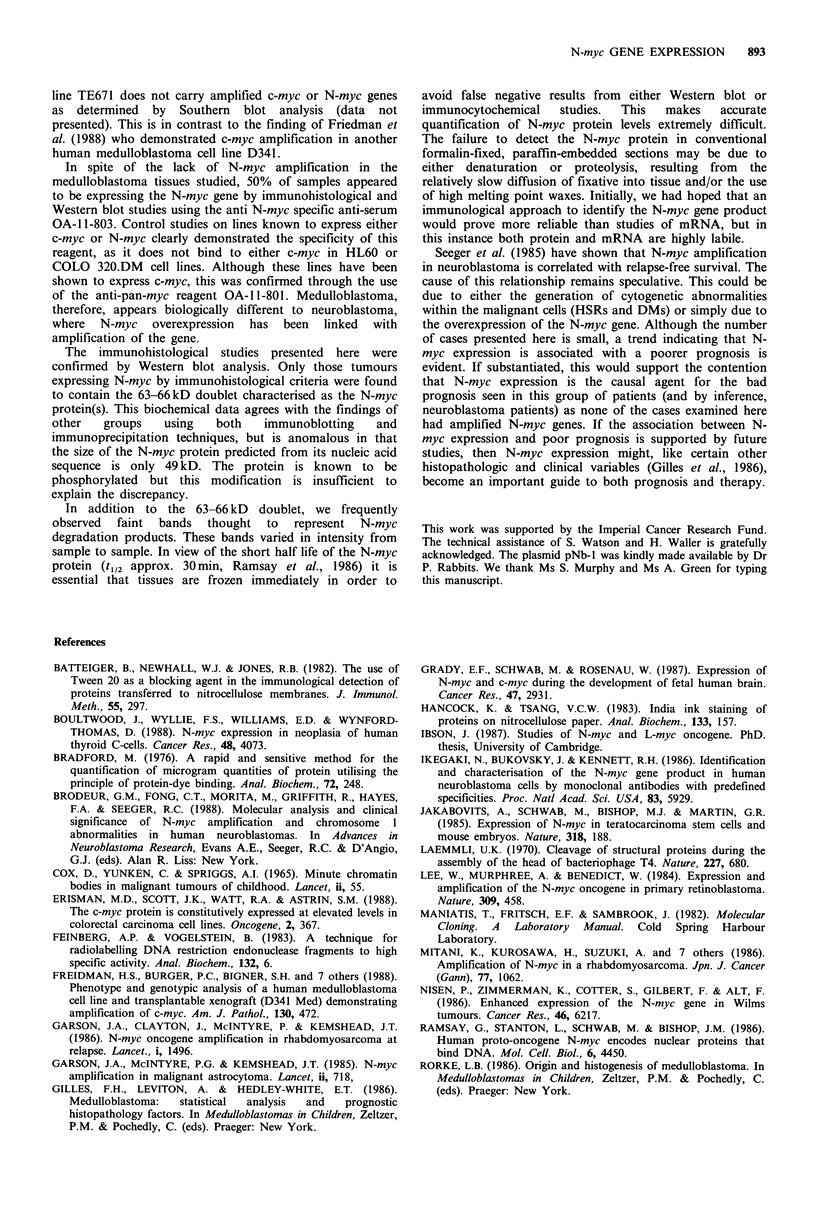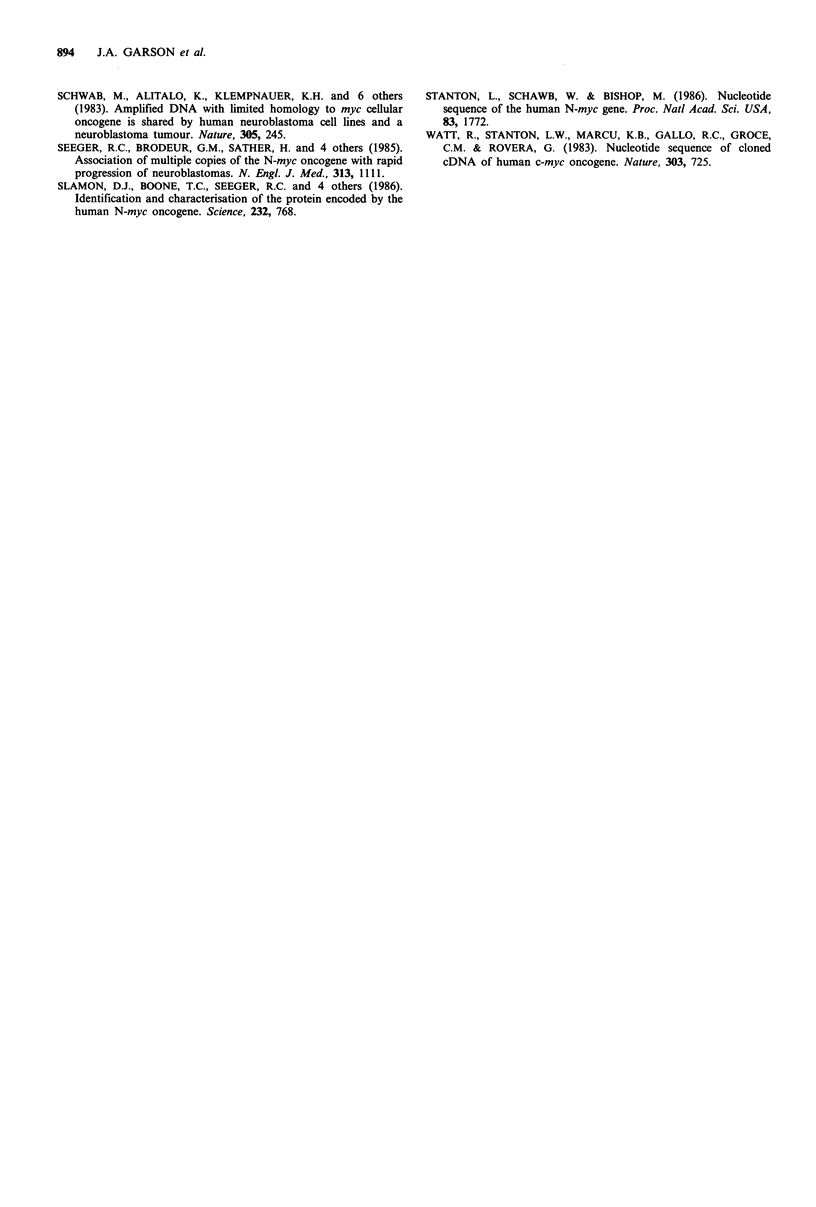# N-myc gene expression and oncoprotein characterisation in medulloblastoma.

**DOI:** 10.1038/bjc.1989.188

**Published:** 1989-06

**Authors:** J. A. Garson, L. F. Pemberton, P. W. Sheppard, I. M. Varndell, H. B. Coakham, J. T. Kemshead

**Affiliations:** Imperial Cancer Research Fund Oncology Laboratory, Institute of Child Health, London, UK.

## Abstract

**Images:**


*Br. J Cancer* (1989), **59**, 889–894 © The Macmillan Press Ltd., 1989

### N-*myc* gene expression and oncoprotein characterisation in medulloblastoma

J.A. Garson[1], L.F. Pemberton[1], P.W. Sheppard[2], I.M. Varndell[2], H.B. Coakham[3] & J.T. Kemshead[1]

[1]*Imperial Cancer Research Fund Oncology Laboratory, Institute of Child Health, 30 Guilford Street, London WC1N 1EH, UK;* [2]*Cambridge Research Biochemicals, Button End, Harston, Cambridge CB2 5NX, UK; and* [3]*Department of Neurosurgery, Frenchay Hospital, Bristol, UK.*

**Summary** Although medulloblastoma and neuroblastoma share many common biological, histological and immunological features, the frequency of N-*myc* amplification differs markedly between the two tumours. In this study, Southern blot analysis revealed that the N-*myc* gene was not amplified in any of the nine medulloblastoma samples analysed. In contrast, over-expression of the gene was found in six of 11 samples as determined by immunocytochemistry and/or Western blot analysis, using an antiserum raised against a synthetic peptide representing a sequence unique to the N-*myc* gene product. The specificity of this reagent was demonstrated by studies on a variety of cell lines expressing N-*myc* and/or c-*myc* oncoproteins. Of the 12 medulloblastoma samples collected over a two-year period and analysed in the course of this project, a trend towards longer disease-free survival was noted in the patients having low levels of the N-*myc* protein in their tumour.

Following the original report of N-*myc* amplification in neuroblastoma, other neuroectoderm derived tumours have been shown either to carry the amplification or to overexpress the gene. Amplification has been noted in a case of a paediatric astrocytoma (Garson *et al.*, 1985) and increased expression reported in both fetal brain (Grady *et al.*, 1987) and retinoblastoma (Lee *et al.*, 1984). Certain non-neuroectoderm derived tumours such as embryonal rhabdomyosarcoma have also been shown to carry amplified sequences of N-*myc* (Garson *et al.*, 1986; Mitani *et al.*, 1986). In addition, specimens of Wilm's tumours and teratomas have been shown to overexpress the N-*myc* gene (Nisen *et al.*, 1986; Jakobovits *et al.*, 1985). These observations, along with the finding that only some 38% of stage III and IV tumours show N-*myc* amplification, have reduced the diagnostic significance of this molecular abnormality (Brodeur *et al.*, 1988). However, information regarding the N-*myc* status of neuroblastoma has been shown to be of prognostic importance. Of particular relevance is the observation that patients with Evans stage II disease, that carry the amplification, have a poorer outcome than those with a single copy of the N-*myc* gene per haploid genome (Seeger *et al.*, 1985). Further information concerning the diagnostic and prognostic importance of N-*myc* would become available if overexpression of the gene was also investigated. Unfortunately, many tumour biopsies do not lend themselves to this type of analysis due to the labile nature of RNA.

Within the past two or three years, immunological approaches to studying the c-*myc* and N-*myc* proteins have become available. Ikegaki *et al.* (1986) used monoclonal antibodies raised against a bacterially expressed lac z fusion protein containing a portion of the N-*myc* sequence to detect nuclear staining in the neuroblastoma cell line IMR5. Similarly, Slamon *et al.* (1986) raised a rabbit antiserum against a bovine growth hormone/N-*myc* fusion protein and reported nuclear staining in cell lines and biopsy samples from tumours known to amplify and overexpress the N-*myc* gene. Using an alternative approach, Ramsay *et al.* (1986) described an antiserum raised against a synthetic peptide representative of a sequence present in the N-*myc* gene. This reagent was reported to detect the N-*myc* protein in standard biochemical procedures, but not to bond to the nuclear protein in immunocytochemical studies.

Medulloblastoma is a tumour of neuroectoderm origin, with some biochemical and immunological features in common with neuroblastoma (Rorke *et al.*, 1986). Only occasional, single case reports of N-*myc* expression in this malignancy have been published previously (Nisen *et al.*, 1986). In addition, c-*myc* amplification has been observed in the medulloblastoma-derived cell line D341 (Friedman *et al.*, 1988). In this paper we detail studies on N-*myc* amplification and expression in 12 medulloblastoma biopsies. Controls using cell lines expressing either the N-*myc* and/or c-*myc* proteins have revealed approximately 50% of the samples analysed overexpress the N-*myc* gene.

### Materials and methods

### *Tissues and sections*

Tissues taken at operation were immediately cut into 2–3 mm blocks, placed into freezing vials and dropped into liquid nitrogen. Cryostat sections (5–6 $\mu$m) were placed on to gelatine coated slides and stained with antisera to the N-*myc* gene product as described below. In addition, Haematoxylin and Eosin sections were prepared to ensure that tissues used for Southern and Western blot analysis consisted of viable tumour. Histological confirmation of the diagnosis was provided by the Department of Neuropathology, Frenchay Hospital, Bristol, United Kingdom.

### *Cell lines*

The human neuroblastoma cell line was a gift from Dr P. Rabbitts, Ludwig Institute, Cambridge, UK, and the medulloblastoma cell line T671 was a gift from Dr Zeltser, UCLA, Los Angeles, USA. These lines, along with the colorectal carcinoma line, COLO 320.DM, were grown at 37°C in a 6% $CO_2$ adherent cultures using RPMI 1640 supplemented with 10% fetal calf serum 100 IU $ml^{-1}$ of penicillin and 100 $\mu g\, ml^{-1}$ of streptomycin.

The T-cell leukaemic line GH1 and the promyelocytic leukaemic line HL60 were grown as suspension cultures in the medium described above. All cell lines were harvested in exponential growth phase for studies in N-*myc* and c-*myc* expression. The Kelly (YK) cell lines carries 100-fold

Correspondence: J.T. Kemshead.
Received 12 October 1988, and in revised form, 1 February 1989.

amplification of the N-*myc* gene and overexpresses N-*myc* RNA (Shwab *et al.*, 1983). The HL 60 and COLO 320.DM cells over-express the c-*myc* gene product (Boultwood *et al.*, 1988; Erisman *et al.*, 1988). The T-cell line GH1 does not express either c-*myc* or N-*myc* as determined by Northern blot analysis (unpublished observation).

*Southern blot analysis*

DNA was prepared from tissues and cell lines using standard techniques (Mantiatis *et al.*, 1982). This was digested with EcoRI and fragments separated by agarose gel electrophoresis. DNA was transferred to gene screen solid support and this was incubated with the N-*myc* probe pNb-1 labelled with $^{32}P$ by the oligo-labelling technique of Feinberg & Vogelstein (1983). After washing the blots at high stringency, autoradiographs were exposed for 48 h on preflashed Kodak XAR-5 film with an intensifier screen. The N-*myc* gene copy number was estimated relative to placental DNA single copy intensity and molecular weights were calculated from lambda Hind III restriction fragment standards.

*Antiserum to the N-*myc *protein*

Antisera were raised to: (1) a synthetic peptide representing an amino acid sequence unique to the N-*myc* gene product (reference: OA-11-803); (2) a synthetic peptide representing an amino acid sequence largely conversed in the N-*myc*, c-*myc* and L-*myc* oncoproteins (reference: OA-11-801).

The sequences are identified and their relative positions in exons 2 and 3 from c-*myc*, N-*myc* and L-*myc* are given in Figure 1. OA-11-803 was affinity purified against the N-*myc* peptide.

*Immunocytochemical staining*

Cryostat sections (6 μm) or cytospin preparations on gelatine coated slides were fixed for 5 min in 3.7% formaldehyde/phosphate buffered saline (PBS). Slides were treated with 2.5% Triton X-100 in PBS for 10 min and non-specific protein binding blocked by incubation with 10% normal rabbit serum (NRS) in PBS for 10 min. Incubation with either affinity purified sheep anti N-*myc* (1:100 dilution of OA-11-803 in PBS/1% NRS) or the pan-*myc* antiserum OA-11-801 (1:100 dilution of OA-11-801 in PBS/1% NRS) was performed overnight at 4°C.

**Figure 1** Sequence of the peptides used to generate the N-*myc* specific and pan-*myc* hetero-antisera. The N-*myc*, c-*myc* and L-*myc* genes consist of three exons. Part of exon 2 and 3 are transcribed and translated to form the relevant *myc* protein. The sequence used to generate the pan-*myc* reagent was taken from a coding region in exon 2. The homology between the N-*myc*, c-*myc* and L-*myc* sequences is illustrated. The sequence used to generate the N-*myc* specific reagent was taken from a coding region in exon 3. The comparative c-*myc*, N-*myc* and L-*myc* sequences are illustrated, indicating the restricted nature of the N-*myc* sequence. c-*myc* sequence (Watt *et al.*, 1983); N-*myc* sequence (Stanton *et al.*, 1986); L-*myc* sequence (Ibson *et al.*, 1987).

Following three washes in PBS, the slides were incubated at room temperature for 1 h with biotin conjugated rabbit anti-sheep Ig (Vector BA 6000) diluted to 1:300 in PBS containing 1% NRS, 1% normal mouse serum and 1% normal human serum. The slides were washed a further three times in PBS and incubated for 30 min with avidin/biotin/peroxidase complex (Dako ABC reagent K355). After washing three times, the chromogen/substrate solution ($0.5\,mg\,ml^{-1}$ diaminobenzidine in PBS with 0.02% $H_2O_2$) was applied for 5–10 min. The reaction was terminated by washing in PBS and slides mounted in Dako 'Glycergel'. Antiserum preincubated with a 100-fold molar excess of the immunising peptide was used as a negative control in all experiments.

*Western blot analysis*

Tissues and cell lines in exponential growth phase were homogenised on ice in extraction buffer containing 10% glycerol, 0.1 M Tris-HCl pH 6.8, 1 mM EDTA, 2% SDS β-mercaptoethanol, 1 mM phenylmethylsulphonylfluoride and $20\,\mu g\,ml^{-1}$ Leupeptin. The lysates were boiled for 3 min and subsequently centrifuged for 15 min at 10,000 *g*. Supernatants were stored at −70°C for up to three weeks. The protein content of the supernatants was estimated by the method of Bradford (1976). Aliquots (50 μg) were loaded on to each lane of an 8% polyacrylamide gel (13 cm × 8 cm × 1.5 mm) and electrophoresis carried out in the presence of SDS (Laemmli, 1970) at 20 V for 16 h. The gel was electroblotted on to 0.1 μm pore size nitrocellulose at 100 V for 5 h at 15°C. Efficiency of transfer was assessed by Coomassie staining of the gel after blotting and by staining one lane of the nitrocellulose filter for total protein, by the Indian ink method of Hancock *et al.* (1983).

Non-specific protein binding sites on the filter were blocked by incubation overnight in PBS/0.05% Tween 20 (Batteiger *et al.*, 1982). Incubation with OA-11-803 (sheep antibody to N-*myc*) or OA-11-801 (sheep antibody to pan-*myc)* antisera diluted 1:200 in PBS/10% normal rabbit serum (or peptide absorbed control) was performed for 90 min at room temperature, followed by three washes, 5 min each, in PBS. The filter was subsequently incubated with either biotinylated or alkaline phosphatase conjugated rabbit anti-sheep Ig, diluted 1:2000 in PBS 10%/NRS for 1 h at RT. When the biotinylated second layer was used, this was followed by a 30 min incubation with Streptavidin conjugated alkaline phosphatase diluted 1:1000 (BRL, Blue Gene Reagent). Colour development was performed in the dark by incubating the strips for 5–30 min in substrate solution. The chromogenic substrate was made up by adding 4.4 μl Nitroblue tetrazolium (NBT) (at $75\,mg\,ml^{-1}$ in 70% dimethylformamide and 3.3 μl 5-bromo-4-chloro-3-indolyl phosphate (BCIP) (at $50\,mg\,ml^{-1}$ in dimethylformamide) to 1 ml of 0.1 M Tris HCl buffer pH 9.5 containing 0.1 M NaCl and 50 mM $MgCl_2$.

### Results

A total of 12 medulloblastoma cases were examined. Due to the limited amount of fresh tissue available, it was not possible to perform all investigations in each case.

*Southern blot analysis*

Following DNA extraction from medulloblastoma tissue and EcoR1 digestion, faint 2 kb bands were identified as hybridising to the N-*myc* probe in all samples analysed ($n = 9$). Figure 2 illustrates six samples (lanes 2–7). These were estimated to be of single copy intensity per haploid genome, as a comparable signal was obtained from human placental DNA (Figure 2, lane 1). As a positive control, an

**Figure 2** Southern blots showing a single copy N-*myc* gene in medulloblastoma tissue. Lane 1, contains 10 μg of placental DNA and shows a 2 kb band hybridised to the N-*myc* probe NB1, single copy intensity. Lanes 2–7, each lane contains 10 μg of DNA from different medulloblastomas. A 2Kb band of single copy intensity hybridised to the N-*myc* probe NB1 is identified in each lane. Lane 8, contains 10 μg of DNA from a stage IV neuroblastoma showing an approximate 50-fold amplification of the N-*myc* gene.

intense 2 kb signal was obtained from DNA isolated from a stage IV neuroblastoma carrying a 50-fold amplification of the N-*myc* gene. Similar experiments using the c-*myc* probe pUCLYXh16 also revealed no amplification of the c-*myc* gene. EcoR1 digests of DNA probed with pUCLYXh16 revealed two bands of 10 and 6 kb of comparable intensity to the signal obtained from human placental DNA (data not presented).

To control for loading errors on the gels, blots were washed and re-proved with the DNA probe L2.30. This binds to a 2.2 kb EcoR1 fragment present as a single copy per haploid genome. Bands of equal intensity were obtained in all tracks analysed.

*N-*myc *gene expression*

The relative level of the N-*myc* protein was determined by a combination of immunocytochemical and biochemical techniques. Immunocytochemistry, using the affinity purified anti N-*myc* specific antiserum (OA-11-803), revealed dense nuclear staining of the Kelly neuroblastoma cell line known to carry a 100-fold amplification and to overexpress the N-*myc* gene. Within this population, there was considerable heterogenity in the staining observed from cell to cell. The N-*myc* gene product was selectively localised to the nucleus, as neither the cytoplasm or nucleoli appeared to bind the reagent (Figure 3a). In contrast, no staining of the Kelly cell line was observed when the antiserum was premixed with a 100-fold molar excess of the immunising peptide (Figure 3b).

The promyelocytic leukaemia cell line HL60 and the colorectal carcinoma cell line COLO 320 DM did not bind OA-11-803, illustrating that the antiserum does not cross react with the c-*myc* protein (Figure 3c). In addition, no binding was observed using the GH1 cell line that does not constitutively express either c-*myc* or N-*myc* (Figure 3d).

Analysis of medulloblastoma tissue sections revealed nuclear staining in 6/10 samples screened (Figure 4). Four samples gave background staining similar to that seen on

**Figure 3** Anti N-*myc* antiserum OA-11-803 binding to cells expressing the N-*myc* protein. (**a**) The human neuroblastoma cell line Kelly containing multiple copies of the N-*myc* gene and elevated levels of N-*myc* mRNA. Binding of OA-11-803 is restricted to the nucleus. (**b**) As (**a**) but using OA-11-803 preincubated with a 100-fold molar excess of the synthetic peptide used as an immunogen. (**c**) Binding of OA-11-803 to the human leukaemic T-cell GH1. (**d**) Binding of OA-11-803 to the promyelocytic cell line HL60. Similar data were obtained for the cell line COLO 320.DM.

tissues such as lymphoma ($n=3$) and tonsil ($n=4$) which is known not to express the N-*myc* gene. Binding of the antiserum in the six positive samples could be blocked by preincubation of the antiserum with an excess of synthetic peptide.

No staining of a bank of formalin-fixed and paraffin-embedded medulloblastoma tissue was observed using either the pan-*myc* or N-*myc* specific antisera, indicating that the N-*myc* protein is either denatured or degraded during fixation.

*Western blot analysis of the N-*myc *protein*

To confirm that antiserum OA-11-803 actually recognises the N-*myc* gene product, medulloblastoma biopsies were homogenised and subjected to Western blot analysis. Figure 5 illustrates a typical result along with controls undertaken to ensure specificity of binding. A 63–66 kD doublet was observed binding to the N-*myc* specific antiserum OA-11-803. This is the normal positions of the N-*myc* protein(s) as determined by polyacrylamide gel electrophoresis. In addition, a 58 kD band was observed (Figure 5, lane A), which is considered to be a putative breakdown product of the N-*myc* gene. The intensity of this band varied from sample to sample. All other bands observed on the gels were due to non-specific binding of the antiserum, as they could not be removed following pre-incubation of OA-11-803 with

**Figure 4** Anti N-*myc* antiserum OA-11-803 binding to frozen sections of medulloblastoma. (**a**) Medulloblastoma tissue showing nuclear staining with antiserum OA-11-803. (**b**) As (**a**) but using OA-11-803 pre-incubated with a 100-fold molar excess of the synthetic peptide used as an immunogen. (**c**) Binding of OA-11-803 to lymphoma tissue.

a 100-fold molar excess of the immunising peptide (Figure 5, lane B). Only the 63–66 kD proteins could be identified when antibody binding was visualised using an alkaline phosphatase rabbit anti-sheep Ig conjugate. However, the signal obtained under these circumstances was weak and, therefore, an additional amplification step was used to enhance photographic recording.

The 63–66 kD bands were only observed in medulloblastoma cell extracts shown to contain the N-*myc* protein by immunocytochemical analysis. In addition, no 63–66 kD doublet was apparent in extracts of the GH1 T-cell line, not expressing either c- or N-*myc* (Figure 5, lane C). As a positive control for these studies, extracts of the neuroblastoma cell line Kelly were also analysed by Western blot. OA-11-803 specifically bound to the 63–66 kD N-*myc* doublet and also showed weak reactivity to the 58 kD protein (Figure 5, track D). Binding to these proteins was again abolished by prior incubation of the antiserum with an excess of synthetic peptide (Figure 5, lanes D and E).

Indirect evidence that the protein being detected in the medulloblastoma tissues was N-*myc* is provided by analysing cell extracts from cell lines (COLO 320.DM and HL60) constitutively expressing the c-*myc* protein. No bnding was observed with OA-11-803, although the pan-*myc* antibody OA-11-801 did bind to the 63–66 kDa c-*myc* doublet (Figure 6).

*Clinical correlates*

Two of the patients in this study died from causes not directly related to their tumour and a third was lost to follow-up. Of the remaining nine cases, five had tumours in which the N-*myc* gene product was identified and four had tumours with no evidence of gene expression. These two groups were well matched for age, site of tumour and degree of tumour resection and yet all patients with no detectable N-*myc* gene expression remain alive and well 8–54 months from the completion of treatment (Table I). In contrast, 3/5 patients with demonstrable constitutive N-*myc* protein levels have died 7–54 months from initial therapy. The other two remain disease-free 36 and 55 months from the end of treatment. While the patient group is too small to be certain of the significance of these observations, there is a trend towards better survival in the group of patients in which the N-*myc* gene product was absent.

**Figure 5** Western blot analysis of the N-*myc* protein in medulloblastoma tissue using an anti-N-*myc* antiserum (OA:11:803). Lane A, illustration of medulloblastoma extract binding antiserum OA-11-803. Lane B, as lane A, but using antiserum OA-11-803 pre-incubated with a 100 fold molar excess of the synthetic peptide used as an immunogen. Lane C, extract from the human neuroblastoma cell line Kelly incubated with OA-11-801 containing a 100-fold molar excess of the synthetic peptide used as an immunogen. Lane D, as lane C but using antiserum OA-11-803. Lane E, extract from the T-leukaemic cell line GH1 showing no 63–66 kD band binding to the antiserum OA-11-803.

### Discussion

Medulloblastomas and neuroblastomas share many common biological, histological and immunological features (Rorke *et al.*, 1986). Both tumour types may exhibit karyotypic abnormalities known as double minute chromosomes (Cox *et al.*, 1965). In neuroblastomas, these, along with homogenously staining regions (HSR), have been shown to be the sites of amplification of the N-*myc* oncogene. Although we have not undertaken cytogenetic studies on the medulloblastomas, reported here by Southern blot analysis, they do not contain either amplified N-*myc* or c-*myc* genes. In addition, in our hands the human medulloblastoma cell

**Figure 6** Western blot analysis of the c-*myc* protein in the promyelocytic cell line HL60. Lane A, extract of the HL60 cell line incubated with the pan-*myc* reagent OA-11-801. Lane B, extract of the HL60 cell line incubated with the N-*myc* specific reagent OA-11-803. Identical results to those illustrated were obtained analysing extracts of COLO 320.DM.

**Table I** Summary of survival data and N-*myc* protein levels in patients with medulloblastoma

| *Patient* | *Age (months)* | *Relapse-free survival (months)* | *Current status* | *N-*myc *copy number* | *N-*myc *protein detected* |
|---|---|---|---|---|---|
| 1 | 10 | 41 | Alive | 1 | – |
| 2 | 30 | 8 | Alive | n.t. | – |
| 3 | 72 | 54 | Alive | 1 | – |
| 4 | 86 | 29 | Alive | 1 | – |
| 5 | 45 | 23 | Dead | 1 | + |
| 6 | 57 | 54 | Dead | 1 | + |
| 7 | 58 | 7 | Dead | 1 | + |
| 8 | 41 | 55 | Alive | n.t. | + |
| 9 | 102 | 36 | Alive | 1 | + |
| 10 | 60 | 0.5 | Dead[a] | 1 | n.t. |
| 11 | 228 | 1 | Dead[b] | 1 | – |
| 12 | n.a. | n.a. | n.a. | n.t. | + |

Patients 10, 11 and 12 were excluded from the summary of clinical data; n.a., data not available; n.t., not tested due to insufficient biopsy material.

[a]Died of post-operative cardiac arrest; [b]Died of bacterial meningitis.

line TE671 does not carry amplified c-*myc* or N-*myc* genes as determined by Southern blot analysis (data not presented). This is in contrast to the finding of Friedman *et al.* (1988) who demonstrated c-*myc* amplification in another human medulloblastoma cell line D341.

In spite of the lack of N-*myc* amplification in the medulloblastoma tissues studied, 50% of samples appeared to be expressing the N-*myc* gene by immunohistological and Western blot studies using the anti N-*myc* specific anti-serum OA-11-803. Control studies on lines known to express either c-*myc* or N-*myc* clearly demonstrated the specificity of this reagent, as it does not bind to either c-*myc* in HL60 or COLO 320.DM cell lines. Although these lines have been shown to express c-*myc*, this was confirmed through the use of the anti-pan-*myc* reagent OA-11-801. Medulloblastoma, therefore, appears biologically different to neuroblastoma, where N-*myc* overexpression has been linked with amplification of the gene.

The immunohistological studies presented here were confirmed by Western blot analysis. Only those tumours expressing N-*myc* by immunohistological criteria were found to contain the 63–66 kD doublet characterised as the N-*myc* protein(s). This biochemical data agrees with the findings of other groups using both immunoblotting and immunoprecipitation techniques, but is anomalous in that the size of the N-*myc* protein predicted from its nucleic acid sequence is only 49 kD. The protein is known to be phosphorylated but this modification is insufficient to explain the discrepancy.

In addition to the 63–66 kD doublet, we frequently observed faint bands thought to represent N-*myc* degradation products. These bands varied in intensity from sample to sample. In view of the short half life of the N-*myc* protein ($t_{1/2}$ approx. 30 min, Ramsay *et al.*, 1986) it is essential that tissues are frozen immediately in order to avoid false negative results from either Western blot or immunocytochemical studies. This makes accurate quantification of N-*myc* protein levels extremely difficult. The failure to detect the N-*myc* protein in conventional formalin-fixed, paraffin-embedded sections may be due to either denaturation or proteolysis, resulting from the relatively slow diffusion of fixative into tissue and/or the use of high melting point waxes. Initially, we had hoped that an immunological approach to identify the N-*myc* gene product would prove more reliable than studies of mRNA, but in this instance both protein and mRNA are highly labile.

Seeger *et al.* (1985) have shown that N-*myc* amplification in neuroblastoma is correlated with relapse-free survival. The cause of this relationship remains speculative. This could be due to either the generation of cytogenetic abnormalities within the malignant cells (HSRs and DMs) or simply due to the overexpression of the N-*myc* gene. Although the number of cases presented here is small, a trend indicating that N-*myc* expression is associated with a poorer prognosis is evident. If substantiated, this would support the contention that N-*myc* expression is the causal agent for the bad prognosis seen in this group of patients (and by inference, neuroblastoma patients) as none of the cases examined here had amplified N-*myc* genes. If the association between N-*myc* expression and poor prognosis is supported by future studies, then N-*myc* expression might, like certain other histopathologic and clinical variables (Gilles *et al.*, 1986), become an important guide to both prognosis and therapy.

This work was supported by the Imperial Cancer Research Fund. The technical assistance of S. Watson and H. Waller is gratefully acknowledged. The plasmid pNb-1 was kindly made available by Dr P. Rabbits. We thank Ms S. Murphy and Ms A. Green for typing this manuscript.

**References**

BATTEIGER, B., NEWHALL, W.J. & JONES, R.B. (1982). The use of Tween 20 as a blocking agent in the immunological detection of proteins transferred to nitrocellulose membranes. *J. Immunol. Meth.*, **55**, 297.

BOULTWOOD, J., WYLLIE, F.S., WILLIAMS, E.D. & WYNFORD-THOMAS, D. (1988). N-*myc* expression in neoplasia of human thyroid C-cells. *Cancer Res.*, **48**, 4073.

BRADFORD, M. (1976). A rapid and sensitive method for the quantification of microgram quantities of protein utilising the principle of protein-dye binding. *Anal. Biochem.*, **72**, 248.

BRODEUR, G.M., FONG, C.T., MORITA, M., GRIFFITH, R., HAYES, F.A. & SEEGER, R.C. (1988). Molecular analysis and clinical significance of N-*myc* amplification and chromosome 1 abnormalities in human neuroblastomas. In *Advances in Neuroblastoma Research*, Evans A.E., Seeger, R.C. & D'Angio, G.J. (eds). Alan R. Liss: New York.

COX, D., YUNKEN, C. & SPRIGGS, A.I. (1965). Minute chromatin bodies in malignant tumours of childhood. *Lancet*, **ii**, 55.

ERISMAN, M.D., SCOTT, J.K., WATT, R.A. & ASTRIN, S.M. (1988). The c-*myc* protein is constitutively expressed at elevated levels in colorectal carcinoma cell lines. *Oncogene*, **2**, 367.

FEINBERG, A.P. & VOGELSTEIN, B. (1983). A technique for radiolabelling DNA restriction endonuclease fragments to high specific activity. *Anal. Biochem.*, **132**, 6.

FREIDMAN, H.S., BURGER, P.C., BIGNER, S.H. and 7 others (1988). Phenotype and genotypic analysis of a human medulloblastoma cell line and transplantable xenograft (D341 Med) demonstrating amplification of c-*myc*. *Am. J. Pathol.*, **130**, 472.

GARSON, J.A., CLAYTON, J., McINTYRE, P. & KEMSHEAD, J.T. (1986). N-*myc* oncogene amplification in rhabdomyosarcoma at relapse. *Lancet.*, **i**, 1496.

GARSON, J.A., McINTYRE, P.G. & KEMSHEAD, J.T. (1985). N-*myc* amplification in malignant astrocytoma. *Lancet*, **ii**, 718,

GILLES, F.H., LEVITON, A. & HEDLEY-WHITE, E.T. (1986). Medulloblastoma: statistical analysis and prognostic histopathology factors. In *Medulloblastomas in Children*, Zeltzer, P.M. & Pochedly, C. (eds). Praeger: New York.

GRADY, E.F., SCHWAB, M. & ROSENAU, W. (1987). Expression of N-*myc* and c-*myc* during the development of fetal human brain. *Cancer Res.*, **47**, 2931.

HANCOCK, K. & TSANG, V.C.W. (1983). India ink staining of proteins on nitrocellulose paper. *Anal. Biochem.*, **133**, 157.

IBSON, J. (1987). Studies of N-*myc* and L-*myc* oncogene. PhD. thesis, University of Cambridge.

IKEGAKI, N., BUKOVSKY, J. & KENNETT, R.H. (1986). Identification and characterisation of the N-*myc* gene product in human neuroblastoma cells by monoclonal antibodies with predefined specificities. *Proc. Natl Acad. Sci. USA*, **83**, 5929.

JAKABOVITS, A., SCHWAB, M., BISHOP, M.J. & MARTIN, G.R. (1985). Expression of N-*myc* in teratocarcinoma stem cells and mouse embryos. *Nature*, **318**, 188.

LAEMMLI, U.K. (1970). Cleavage of structural proteins during the assembly of the head of bacteriophage T4. *Nature*, **227**, 680.

LEE, W., MURPHREE, A. & BENEDICT, W. (1984). Expression and amplification of the N-*myc* oncogene in primary retinoblastoma. *Nature*, **309**, 458.

MANIATIS, T., FRITSCH, E.F. & SAMBROOK, J. (1982). *Molecular Cloning. A Laboratory Manual.* Cold Spring Harbour Laboratory.

MITANI, K., KUROSAWA, H., SUZUKI, A. and 7 others (1986). Amplification of N-*myc* in a rhabdomyosarcoma. *Jpn. J. Cancer* (*Gann*), **77**, 1062.

NISEN, P., ZIMMERMAN, K., COTTER, S., GILBERT, F. & ALT, F. (1986). Enhanced expression of the N-*myc* gene in Wilms tumours. *Cancer Res.*, **46**, 6217.

RAMSAY, G., STANTON, L., SCHWAB, M. & BISHOP, J.M. (1986). Human proto-oncogene N-*myc* encodes nuclear proteins that bind DNA. *Mol. Cell. Biol.*, **6**, 4450.

RORKE, L.B. (1986). Origin and histogenesis of medulloblastoma. In *Medulloblastomas in Children*, Zeltzer, P.M. & Pochedly, C. (eds). Praeger: New York.

SCHWAB, M., ALITALO, K., KLEMPNAUER, K.H. and 6 others (1983). Amplified DNA with limited homology to *myc* cellular oncogene is shared by human neuroblastoma cell lines and a neuroblastoma tumour. *Nature*, **305**, 245.

SEEGER, R.C., BRODEUR, G.M., SATHER, H. and 4 others (1985). Association of multiple copies of the N-*myc* oncogene with rapid progression of neuroblastomas. *N. Engl. J. Med.*, **313**, 1111.

SLAMON, D.J., BOONE, T.C., SEEGER, R.C. and 4 others (1986). Identification and characterisation of the protein encoded by the human N-*myc* oncogene. *Science*, **232**, 768.

STANTON, L., SCHAWB, W. & BISHOP, M. (1986). Nucleotide sequence of the human N-*myc* gene. *Proc. Natl Acad. Sci. USA*, **83**, 1772.

WATT, R., STANTON, L.W., MARCU, K.B., GALLO, R.C., GROCE, C.M. & ROVERA, G. (1983). Nucleotide sequence of cloned cDNA of human c-*myc* oncogene. *Nature*, **303**, 725.